# Aboveground biomass estimation in a grassland ecosystem using Sentinel-2 satellite imagery and machine learning algorithms

**DOI:** 10.1007/s10661-024-13610-1

**Published:** 2025-01-06

**Authors:** Andisani Netsianda, Paidamwoyo Mhangara

**Affiliations:** 1 School of Geography, Archaeology and Environmental Studies, University of the Witwatersrand, Johannesburg, 2000 South Africa; 2 Council for Geosciences, 280 Pretoria Street, Pretoria, 0184 Silverton South Africa

**Keywords:** Carbon stock, Leaf area index, Vegetation indices, Carbon cycle, Carbon sink

## Abstract

The grassland ecosystem forms a critical part of the natural ecosystem, covering up to 15–26% of the Earth’s land surface. Grassland significantly impacts the carbon cycle and climate regulation by storing carbon dioxide. The organic matter found in grassland biomass, which acts as a carbon source, greatly expands the carbon stock in terrestrial ecosystems. Correct estimation of above ground biomass (AGB) and its spatial and temporal changes is vital for determining the carbon cycle of the grassland. Datasets from multiple sources were fused to accomplish the objective of the study. The Sentinel-2 sensor band, vegetation index (NDVI), and Shuttle Radar Topography Mission (SRTM) DEM products were used as predictor variables, while Global Ecosystem Dynamics Investigations (GEDI) mean above-ground biomass density (AGBD) data was used to train the model. Random forest (RF) and gradient boosting were used to estimate the AGB of the grassland biome. We also identified the correlation between Sentinel-2-derived vegetation indices and ground-based measurements of leaf area index (LAI). The processing duration, parameter requirements, and human intervention are reduced with RF and gradient boosting algorithms. Due to its fundamental concept, ensemble algorithms effectively handled multi-modal data and automatically conducted spectral selection. The findings show variations in the study area’s AGB concentration throughout five years. According to the results, gradient boosting models outperformed RF models in both years. RF achieved the highest *R*^2^ value of 0.5755 Mg/ha, while gradient boosting achieved the highest *R*^2^ value of 0.7298 Mg/ha. Sentinel-2-derived VI vs LAI results show that NDVI was the best-performing model with an *R*^2^ value of 0.6396 m^2^ m^−2^ and an RMSE of 0.159893 m^2^ m^−2^, followed by OSAVI, NDRE, and MSAVI. This result shows that sensor data and field biophysical data can map the terrestrial ecosystem’s biomass.

## Introduction

The grassland ecosystem forms a critical part of the natural ecosystem, covering up to 15–26% of the Earth’s land surface. Grassland significantly impacts the carbon cycle and climate regulation by storing carbon dioxide. The organic matter found in grassland biomass, which acts as a carbon source, dramatically expands the carbon stock in terrestrial ecosystems (Huang et al., 2022). Aboveground biomass (AGB) can be defined as the mass of living and dead plant tissue above the ground. AGB is a significant indicator of grassland health status and ecosystem function. AGB is greatly vulnerable to changes in climate, human activities, and other disturbances (Shi et al., 2021). Appropriate and correct tracking of AGB modifications in grasslands is of incredible importance for keeping the terrestrial atmosphere in shape and function, maintaining the stability of plant productivity and surface energy, and tracking the movement of carbon on a global scale.

AGB can be estimated using field sampling and remote sensing (RS) (Shi et al., 2021). The traditional method is field-based and may involve cutting vegetation. It is very accurate, but it can damage the vegetation. It is strenuous, time-consuming, and not economical, primarily when the investigation is conducted over a broad area. Thus, remote sensing (RS) technologies offer economical and time-series biomass investigation opportunities (Pang et al., 2020). RS data can be acquired using multispectral and hyperspectral sensors. Although hyperspectral sensors offer high-spatial and spectral resolution benefits, they are expensive, have a narrow width, and are not feasible to apply over a broad area (Pang et al., 2020). Sentinel-2 is a non-commercial multispectral sensor launched by the European Space Agency (ESA) for the first time in 2015. Unlike the Landsat sensor, Sentinel-2 has high spatial and spectral resolution and a revisit time of five days on a global scale (Naidoo et al., 2019). Information from the Sentinel-2 spectral bands can be beneficial in estimating AGB. The red-edge band found on the Sentinel-2 spectrum, known for producing highly correct vegetation biomass estimates, is an example of a helpful band (Naidoo et al., 2019). The spectrum has a red edge between 680 and 750 nm (Mutanga and Skidmore, 2007). To avoid biomass estimation errors and associated costs, relevant RS data for estimating AGB must be selected. According to a study by Houghton, Hall, and Goetz (2009), many researchers have estimated biomass. Still, they find it challenging to determine biomass because in situ data is insufficiently reliable accurately.

This study employed random forest (RF) and gradient boosting machine learning algorithms, to simulate AGBD. RF is a collective learning technique that uses several decision trees. The processing duration, parameter requirements, and human intervention are reduced with RF. Due to its fundamental concept, RF can also effectively handle multi-modal data and automatically conduct spectral selection (Nasiri et al., 2022). Like RF, gradient boosting is an ensemble algorithm that uses decision trees to train the model. They share common traits; however, the latter employs a random selection, and the former builds the decision trees in stages and uses all the trees to generate a strong regressor (Banerjee, 2022). Sentinel-2 sensor spectral bands are fussed with other datasets such as Shuttle Radar Topography Mission (SRTM), elevation, and slope data to improve performance and increase the reliability of the machine learning algorithms models (Shendryk, 2022). The launch of GEDI aided research concerning AGB because it has reduced the work of manually collecting AGB in the field, which usually involves harvesting and measuring tree parameters such as height and volume. The manual acquisition of AGBD data damages the vegetation. GEDI maps the Earth’s structure using a laser at a very high resolution. This accurate measurement of vegetation parameters, canopy height, and elevation has made it possible to study carbon dynamics (Shendryk, 2022). The aboveground biomass density (AGBD) obtained from Global Ecosystem Dynamics Investigation (GEDI) is used as training data. Digital elevation models (DEM), on the other hand, provide critical information; they can measure the height of different objects on the surface of the Earth. This data type is crucial in various fields; it can benefit geomorphological studies, environmental studies, climatological modeling, and agricultural research. In RS, DEM information is essential for providing helpful information about vegetation height. It is useful for figuring out how much carbon is stored in a terrestrial environment (Lei et al., 2021). Research conducted by Shendryk (2022) demonstrates a fusion of various datasets such as GEDI, elevation, Sentinel-1 and −2, and land cover. The objective of mapping AGB by integrating various datasets and feeding them into an ML technique was accomplished by producing AGB maps. The coefficient of determination *R*^2^ and RMSE were used to measure the accuracy of the AGB models, achieving high accuracy, an *R*^2^ of 0.66–0.74 and an RMSE of 55–81 Mg/ha. Challenges also exist in sensor data; for example, GEDI has geolocation faults. However, this can be solved by filtering the data.

This study also used vegetation indices (VIs) from Sentinel-2 sensor imagery. On the electromagnetic spectrum, the VIs are developed or derived from the visible and near-infrared (NIR) bands (red, green, and blue bands) (Shi et al., 2021). Narrow bands vegetation indices are significant because they can offer crucial details on plants’ chemical and biophysical properties, according to research from earlier studies (Darvishzadeh et al., 2008). The normalized difference vegetation index (NDVI) is an example of VI and is used most frequently. However, it is sensitive to terrain that is not entirely covered by vegetation, and this is where modified soil-adjusted vegetation index (MSAVI) and optimized soil-adjusted vegetation index (OSAVI) come in as they are suitable indexes for overcoming this effect (Wang et al., 2022). The VI values range from − 1 to 1. Low values indicate rocky outcrops and barren soil. Medium values indicate the presence of grassland and shrubs, while high values closer to 1 indicate dense vegetation or forest, see Table 4 (Muavhi, 2021). The VIs can be combined with LAI to assess their statistical correlation. The biophysical parameter (LAI) is vital in photosynthetic, energy balance, and transpiration processes on the Earth’s land surface. Previous studies have indicated that information obtained in the red, red-edge, green, and near-infrared bands correlates well with LAI (Masemola et al., 2016). Pang et al. (2020) also indicated the significant correlation that exists between these indices and biomass. The main objective of this study is to estimate the AGB of the grassland biome by integrating datasets from multiple sources, to compare the performance of random forest and gradient boosting methods and to identify the correlation between Sentinel-2-derived vegetation indices and ground-based measurements of LAI.

## Materials and methods

### Study area location

**Fig. 1:**
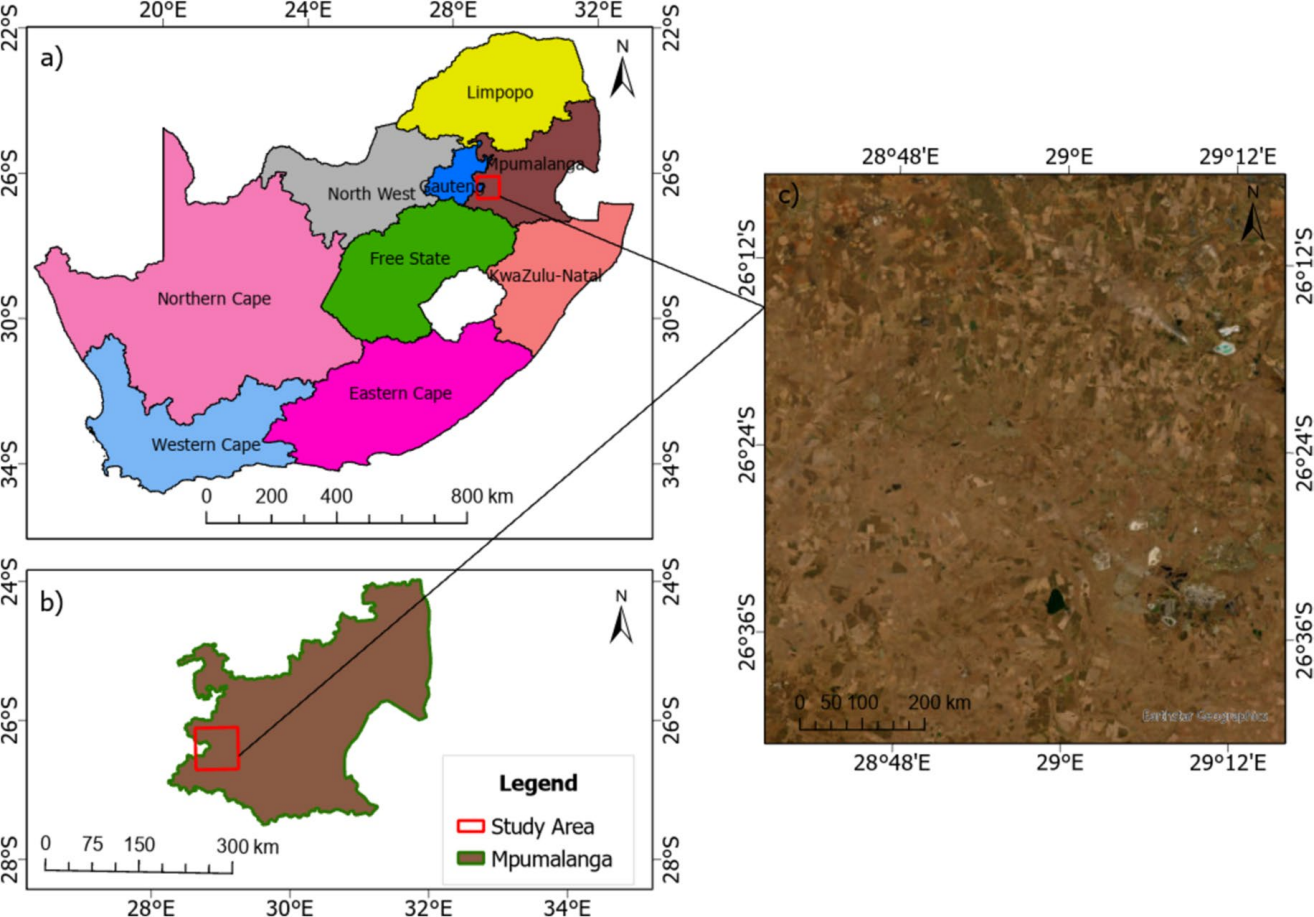
The study area location: **a** shows the study areas at a national level, **b** shows the study area at a provincial level, and **c** shows the study area at a local level (the white strokes on the online Arc imagery are gases from the power station)

**Fig. 2:**
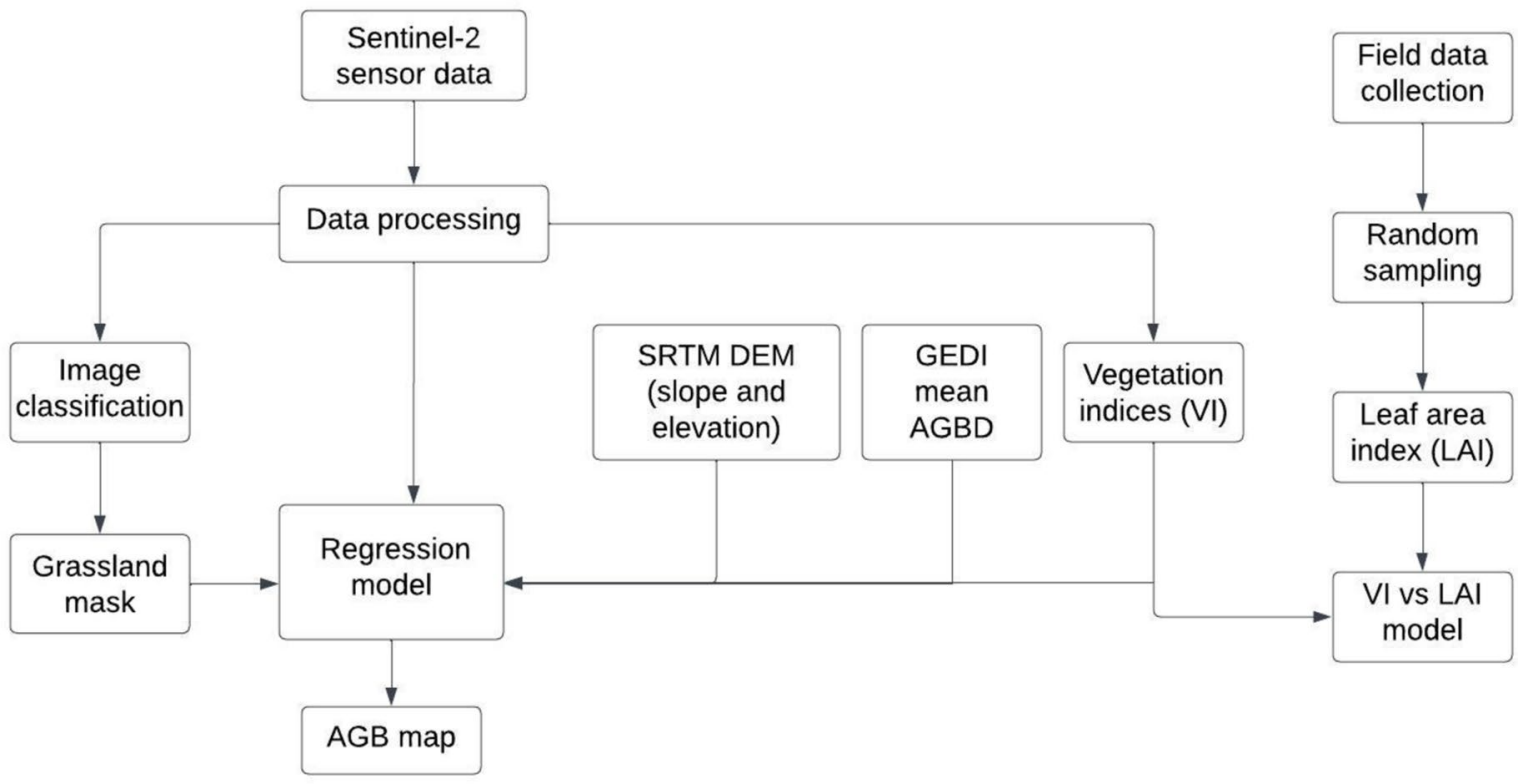
Methodological flowchart

Grassland covers about 26% of South Africa’s land. The South African grassland biome faces extinction challenges as approximately 45% of it is being transformed for other land uses such as agriculture, mining, and built-up areas (Naidoo et al., 2019). The study area lies within Leandra farming town, north-west (− 26.268 to − 26.467 and 28.656 to 29.253) of Secunda town, and it falls within the Govan Mbeki municipality in the Mpumalanga province of South Africa (Fig. 1). It is characterized by flat terrain and an elevation of approximately 1600 m above sea level (Cadman et al., 2013). The area was initially established for agricultural purposes and later expanded owing to coal and gold mining activities. The southeast section of the study area has extensive mine tailings from the Winkelhaak Mines and Evander and Embalenhle settlements. The climate of this area is characterized by summers that are warm and wet and winters that are dry and cold. The average annual rainfall received in this area ranges from 750 to 900 mm per year (Naidoo et al., 2019). The vegetation in this area is found within the grassland biome, and the vegetation type is highveld grassland (Cadman et al., 2013). Mining activities, urban development, and agriculture threaten the South African grassland biome (Fourie et al., 2015). Figure 1 shows the location of the study area on a national, provincial, and local scale. Figure 2 shows the methodological flowchart implemented in this current study. It shows the various datasets, data processing steps, data analysis procedures, and methods used to measure model performance.

### Data collection

The data utilized in this study can be downloaded for free on their respective websites. All sensor data used was accessed through the Google Earth Engine (GEE) platform. These datasets included Sentinel-2 level 2A data, a product of Copernicus, Shuttle Radar Topography Mission (SRTM) digital elevation model (DEM) data, a product of National Geospatial-Intelligence Agency and NASA collaboration, Global Ecosystem Dynamics Investigations (GEDI) mean aboveground biomass density (AGBD) data a product of NASA as well as the land cover maps produced from Sentinel-2 images within the same GEE platform (Shendryk, 2022).

#### Sentinel-2 data

Sentinel-2 is a multispectral sensor responsible for the collection of RS data. The ESA first launched the Sentinel-2 sensor in 2015. Sentinel-2 is composed of two sensors, which are Sentinel-2A and Sentinel-2B. Sentinel-2A was launched on June 23, 2015, while Sentinel-2B was launched on March 7, 2017. The sensor is classified as a medium-resolution sensor. The Sentinel-2 sensor has 13 observation bands distributed throughout the electromagnetic (EM) spectrum, from the visible to the SWIR, with a spatial resolution of 10, 20, and 60 m. Four Sentinel-2 bands are found in the VNIR portion of the EM spectrum, five in the NIR portion of the EM spectrum, and the remaining three in the SWIR portion of the EM spectrum. These two sensors have the same orbit and a 5-day revisit when combined. Sentinel-2 sensor characteristics have made it very popular to monitor vegetation changes, detect land cover changes, and manage natural catastrophes (Nguyen et al., 2020). Table 1 shows the detailed characteristics of the Sentinel-2 sensor. In this study, 8 Sentinel-2 Level-2A products were used (Table 2). Level-2A symbolizes that the satellite images accessed already contained atmospheric, geometric, and radiometric corrections, and as a result, no further corrections were applied. Sentinel-2 data can be accessed freely through the USGS Earth Explorer and the ESA Copernicus Open Access Hub (Vajsová et al., 2020). However, for this study, the sensor data is accessed through the Google Earth Engine (GEE) web platform (Liang et al., 2023).

**Table 1:** Description of Sentinel-2 sensor characteristics

Band name	Spatial resolution	Wavelength	Band description	Revisit	Data availability
B1 B2 B3 B4 B5 B6 B7 B8 B8A B9 B10 B11 B12	60 m 10 m 10 m 10 m 20 m 20 m 20 m 10 m 20 m 60 m 60 m 20 m 20 m	443 nm 490 nm 560 nm 665 nm 705 nm 740 nm 783 nm 842 nm 865 nm 940 nm 1375 nm 1610 nm 2190 nm	Aerosols Blue Green Red Red-edge A Red-edge B Red-edge C NIR Red-edge D Water vapor Cirrus SWIR SWIR	Five days with Sentinel-2A and 2B, and 10 days with a single sensor	2015

**Table 2:** Description of image collection

Sensor	Processing level	Number of image collection	Tile number	Acquisition date
Sentinel-2	S2_SR_HARMONIZED	2 2 2 2	T35JPL T35JPM T35JQL T35JQM	20190226, 20230111

#### GEDI data

The Global Ecosystem Dynamics Investigations (GEDI) mean aboveground biomass density (AGBD) data is used to estimate AGB. The fundamental objective of the GEDI sensor is to map the AGBD of the terrestrial ecosystem remotely (Duncanson et al., 2022). With a complete waveform LIDAR system controlled by the International Space Station (ISS), GEDI can record measurements of vegetation on the Earth’s surface. The GEDI sensor was established on December 5, 2018, and only acquired data in April 2019 (Shendryk, 2022). GEDI is a product of NASA. It maps the Earth’s structure using a laser at a very high resolution. This accurate measurement of vegetation parameters, canopy height, and elevation has made it possible to study carbon dynamics (Shendryk, 2022). Even though the GEDI products are valuable for AGB calculations, there are many issues with their geolocation faults. This issue can be reduced by filtering the data. The GEDI L4B product gives mean AGBD estimations for a 1 km by 1 km area. GEDI AGBD is measured in megagrams per hector (mg/ha) (Shendryk, 2022). GEDI sensors map the Earth’s land surface at latitudes between 51.6° north and 51.6° south at a near-global scale. This data is freely available (Liang et al., 2023).

#### SRTM DEM data

This study used SRTM DEM with a spatial resolution of one arc second, equivalent to 30 m on the ground. The SRTM DEM was used to obtain elevation and slope information. The SRTM was established by collaborating with the National Geospatial-Intelligence Agency, the National Aeronautics and Space Administration (NASA), and German and Italian space agencies. SRTM produces DEM data between 60° north latitude and 56° south latitude; it covers almost the entire globe. It was created using information gathered over 11 days at the beginning of 2000 by specially designed radar equipment aboard the Space Shuttle Endeavour (SSE). The SRTM DEM data is open source and can be accessed through (https://lpdaac.usgs.gov/products/srtmimgmv003/). However, for this study, the data was accessed through the GEE platform, and the spatial resolution of the slope and elevation remained unchanged at 30 m. The slope and the elevation were reprojected to WGS 1984 UTM zone 35 s to match Sentinel-2 data already in a similar projection (Nasiri et al., 2022).

#### LAI measurements

**Fig. 3:**
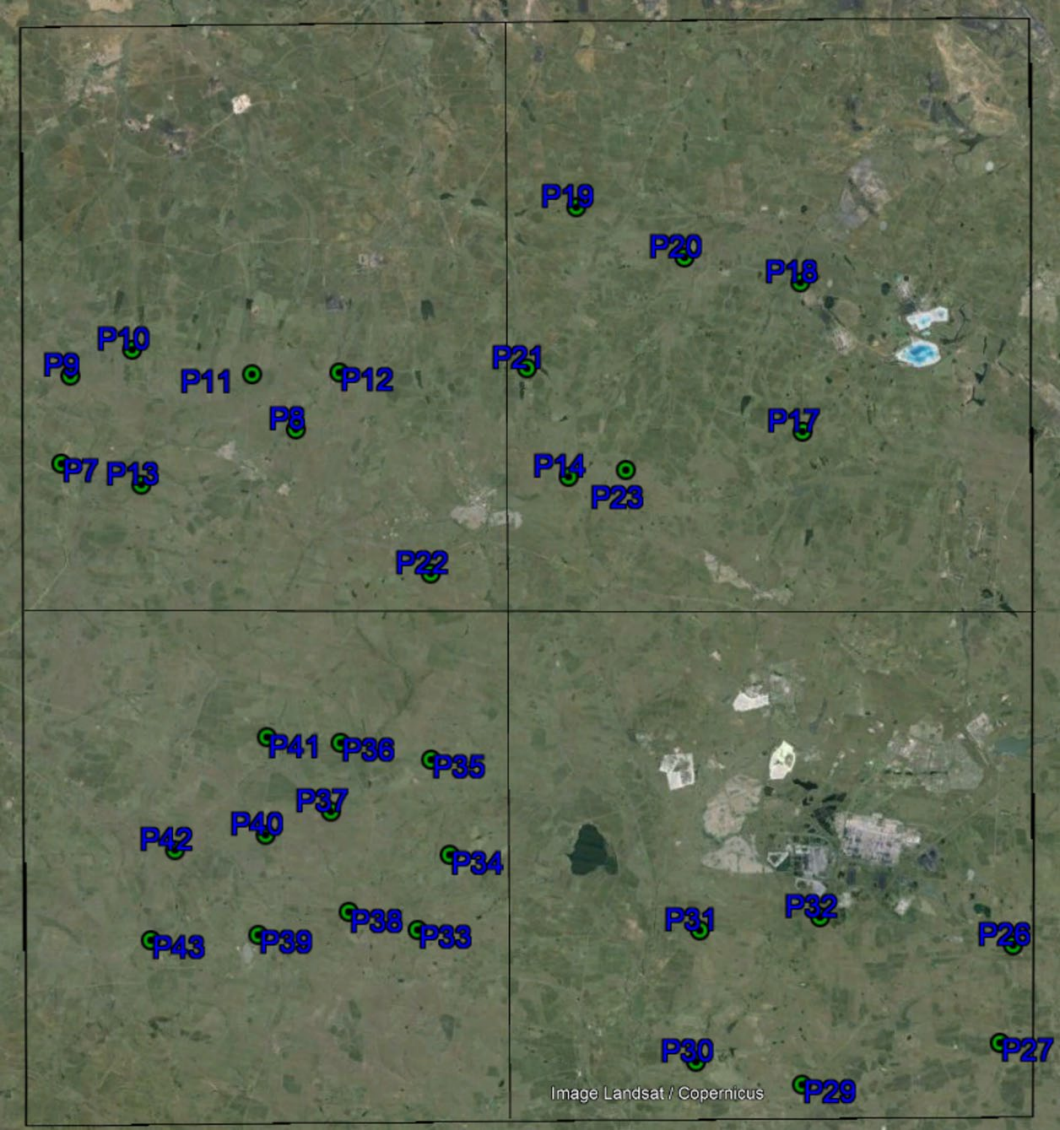
Field sample locations are overlaid on Google’s Image

The biophysical parameter LAI is vital in photosynthetic, energy balance, and transpiration processes on the Earth’s land surface. Previous studies have indicated that information obtained in the red, red-edge, green, and near-infrared bands correlates well with LAI (Masemola et al., 2016). The LI-COR LAI-2200C plant canopy analyzer, a product of Biosciences in the USA, was used to measure LAI (Danner et al., 2015). The data was gathered in May of 2023 on a 405 000 hectares grid. The LI-COR LAI-2200C plant canopy analyzer was placed above and below the canopy for each point measured every 5 km where possible. Three measurements were observed above and below the canopy, and an average LAI was obtained. The LAI measurements were taken during consistent sky illumination, facing away from the sun to avoid direct sunlight on the LI-COR LAI-2200C plant canopy analyzer, and the operator avoided the sensor’s view (Cho & Ramoelo, 2016). The study area was divided into four plots. A random stratified sampling technique was used in each plot to guarantee that the sampled areas would accurately represent the plot. Forty-three samples were collected; however, some sample points were discarded due to uncertainty within them, leaving 32 samples as the total of samples analyzed in Fig. 3. This explains why some areas within the plots do not have sample points. LAI was recorded in m^2^ m^−2^ unit measurement. The lowest sample reading was 3.44 m^2^ m^−2^, and the highest was 4.49 m^2^ m^−2^ (Shafian et al., 2018). A Garmin handheld global positioning system (GPS) was used alongside LI-COR LAI-2200C to collect the coordinates of 33 reference data points. Garmin GPS has a horizontal accuracy of + / − 5 m (Darvishzadeh et al., 2008). Figure 3 shows an overview of ground samples of LAI.

### Data processing

#### Image downloading and pre-processing

To enhance the quality of the products, instead of using all 13 bands of the Sentinel-2 sensor, this study used nine bands: bands 2, 3, 4, 5, 6, 7, 8, 11, and 12 (Darvishzadeh et al., 2008). All images used in this study were accessed through the Google Earth Engine (GEE) platform—the ee.ImageCollection (“COPERNICUS/S2_SR_HARMONIZED”) image collection code was used to download Sentinel-2 images within the GEE platform. To produce cloud-free images, a code for masking the clouds was implemented using the Sentinel-2 QA60 band that contains the clouds and the cirrus at 10 and 11 bits, respectively. Only cloud-free images of specific dates were downloaded. The images were resampled to 10 m pixel resolution to enhance the visibility of features on the Earth’s surface. Only cloud-free images of specific dates were downloaded. The filtered scenes were mosaicked using the mosaic function. All image processing is done on the GEE platform (De Luca et al., 2022).

#### Computing vegetation indices from Sentinel-2 satellite data

On the electromagnetic (EM) spectrum, vegetation indices (VIs) are developed or derived from the visible to near-infrared (NIR) bands (red, green, blue bands, and red edge) that have a wavelength range of between 442,7 nm to 864.nm (Shi et al., 2021). The VIs were computed using GEE scripts. These VIs are used because of their ability to map vegetation yield. Narrow-band vegetation indices are significant because they can offer crucial details on plants’ chemical and biophysical properties, according to research from earlier studies (Darvishzadeh et al., 2008). The normalized difference vegetation index (NDVI) is frequently used index. However, it has a saturation effect. To account for this effect, enhanced VIs that can overcome the limitations of NDVI are adopted (Wang et al., 2022). The following enhanced VIs were used: modified soil-adjusted vegetation index (MSAVI2), normalized difference red-edge index (NDRE), and optimized soil-adjusted vegetation index (OSAVI) (Table 3) (Shi et al., 2021). All the VIs follows the same principle of using two band combinations. However, OSAVI and MSAVI2 include soil factors. The development of vegetation indices involves the mathematical fusion of distinct EM spectral bands. These indices are associated with canopy characteristics that might improve vegetation signals by mitigating the influences of soil and atmosphere (Hussain et al., 2020). The VI values range from − 1 to 1. Low values indicate rocky outcrops and barren soil. Medium values indicate the presence of grassland and shrubs, while high values closer to 1 indicate dense vegetation or forest, see Table 4 (Muavhi, 2021).

**Table 3:** Formula for vegetation index

Vegetation indices	Formula
NDVI	$\frac{\text{NIR}-\text{red}}{\text{NIR}+\text{red}}$
OSAVI	$\left(1+\text{L}\right)\frac{\text{NIR}-\text{red}}{\text{NIR}+\text{red}}$
NDRE	$\frac{\text{NIR}-\text{red edge}}{\text{NIR}+\text{red edge}}$
MSAVI2	$\text{L }\times (\left(2\times \text{NIR}+1\right))-\sqrt{\left(2\times \text{NIR}+1\right)^2-8\times (\text{ NIR}-\text{Red})}$

**Table 4:** Object and corresponding NDVI values

Object on surface	NDVI values
Waterbodies	− 1
Bare soil	0–0.2
Shrubs and grassland	0.2–0.5
Dense vegetation and forest	< 0.5

#### Machine learning algorithm for estimating aboveground biomass

Machine learning (ML) is a branch of artificial intelligence. ML can automate the prediction process, making it very attractive to researchers in a wide range of fields. ML takes advantage of new datasets to continuously enhance its learning. It is a reliable technique for identifying trends and patterns in the data (Banerjee, 2022). The random forest (RF) and gradient boosting techniques were used to perform regression in this study.

Gradient boosting is a machine learning algorithm that is used to construct a regression model. It is an ensemble learning approach in which every tree used in the various building phases aims to accomplish the same objective. The trees used in the initial stages of construction are strengthened by adding more trees. This process implemented to overcome the hurdles of existing weak learners, and it continues until the development of a strong leaner. The parameters used to construct this model includes the number of trees which are referred to as learners, we also used the shrinkage parameter for controlling the rate of learning, we used the default stochastic tree boosting sampling rate. The least squares loss function was used to assess the behavior of the tree leaners. Choosing the right parameters for the model assisted in speeding up processing and lessen the impact of overfitting (Banerjee, 2022).

Regression trees are also used to accomplish learning in random forest, another kind of ensemble approach. This method uses the similar approach to that of gradient boosting; however, instead of using the entire volume of constructed regression trees, a random selection takes place. The random selection reduces the discrepancy in the data, while enhancing the model’s accuracy. The next stage minimizes contamination by selecting an appropriate regressor and a suitable splitting value (*mtry*). The parameters used include number of trees (*ntree*), variable per split (*mtry*), minimum leaf population, seed, and a default bag fraction of 0.5. The parameters specified above ascertain high levels of accuracy in the random forest model (Banerjee, 2022). Due to its fundamental concept, RF can also effectively handle multi-modal data and automatically conduct spectral selection (Nasiri et al., 2022).

The training data derived on the GEDI L4B dataset amounts to 966 points for each model. The training data is 671, which accounts for 70% of the overall data, and the validation data is 295 points which accounts for 30% of the data. The *R*^2^, which is the coefficient of determination, and the RMSE were calculated to determine the performance of the models (Naidoo et al., 2019).

#### Variable significance for the RF model

The term “variable significance” refers to the variables’ role in predicting the model, which enhances model accuracy while eliminating unnecessary data and reducing processing time. This research used the variable of significance approach to determine the contribution of each variable to the model (Nasiri et al., 2022). This exercise was carried out in this research by initially incorporating all predictor variables into the model, thereby eliminating the least-performing variable. This procedure was implemented until all the least important variables were eliminated. Examples of predictor variables included Sentinel-2 spectral bands, SRTM DEM products, and the traditional vegetation index NDVI (Shah et al., 2020).

#### Statistical analysis of validation data

The validation of the AGB model relied on independent evaluation data. Entirely distinct independent evaluation data is necessary to ensure the model’s reliability (Darvishzadeh et al., 2008). The LAI data collected from the field was used to evaluate the accuracy of the AGB maps. This was done by using mathematical formulas to assess the correlation between LAI and VIs derived from Sentinel-2 imagery. The following VIs, NDVI, MSAVI2, NDRE, and OSAVI, were the independent variables, and the LAI was the dependent variable (Zhang et al., 2018). The bands used to compute the VIs can be located between the visible and near-infrared bands, referred to as narrow bands of the EM spectrum. Prior research has demonstrated that VIs derived from the narrow bands carry potential information for quantifying the biophysical properties of vegetation. The linear regression approach was used to relate VIs with the biophysical characteristics of the grass canopy. We resampled Sentinel-2 imagery to 10 m spatial resolution. The study area is 405 000 hectors in size. We planned to sample every 5 km but due to access restrictions some data points were as far as 8 km apart. Locations of LAI data collected in the field were used to extract vegetation indices data. Parameters such as the RMSE and the *R*^2^ were used in this study to assess the performance of the developed models (Zhang et al., 2018). These statistical indicators require the data to be divided into training and validation sets. Models created using the training data set are used to forecast the response factor from the evaluation data set (Darvishzadeh et al., 2008).

## Results

### Aboveground biomass

**Fig. 4:**
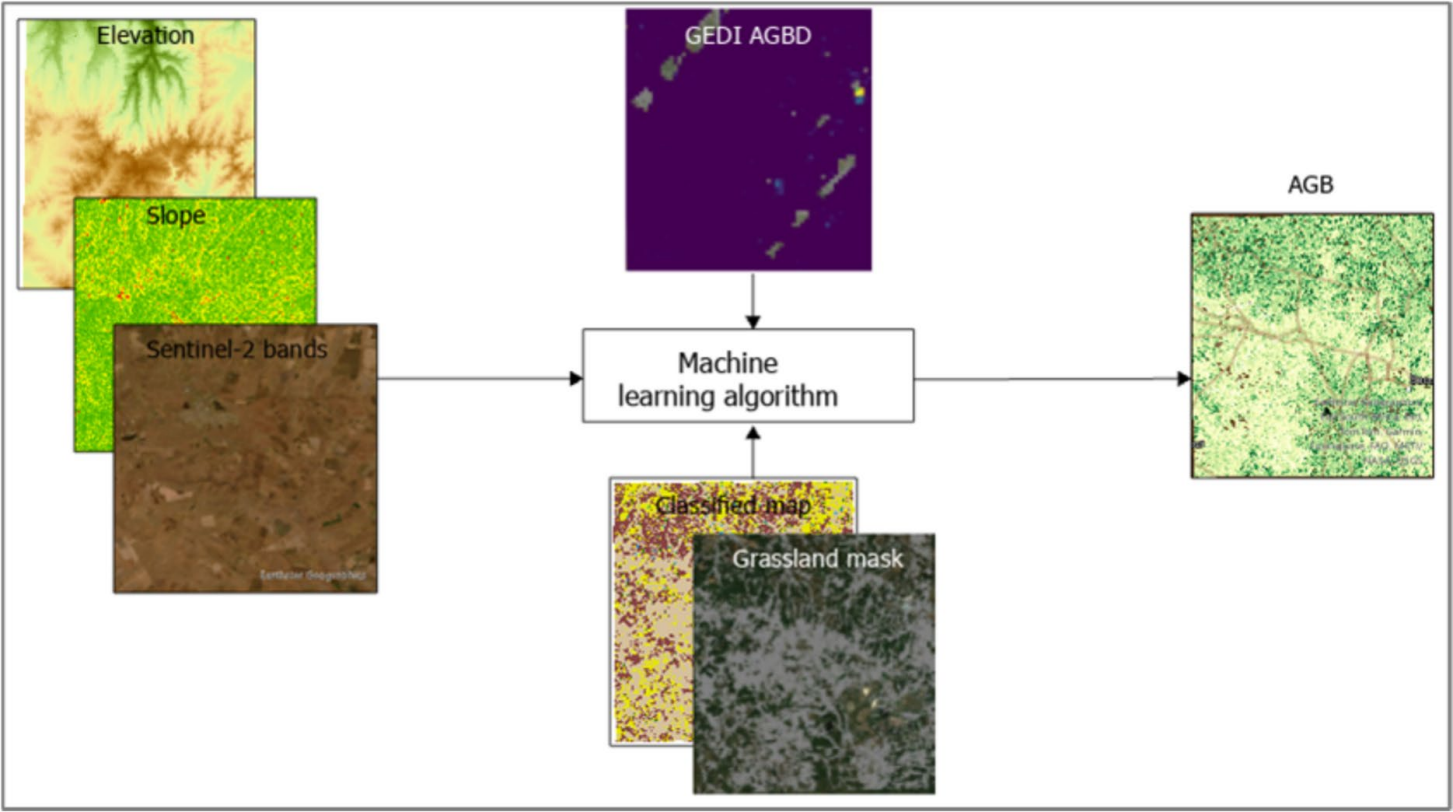
Process for mapping AGB

**Fig. 5:**
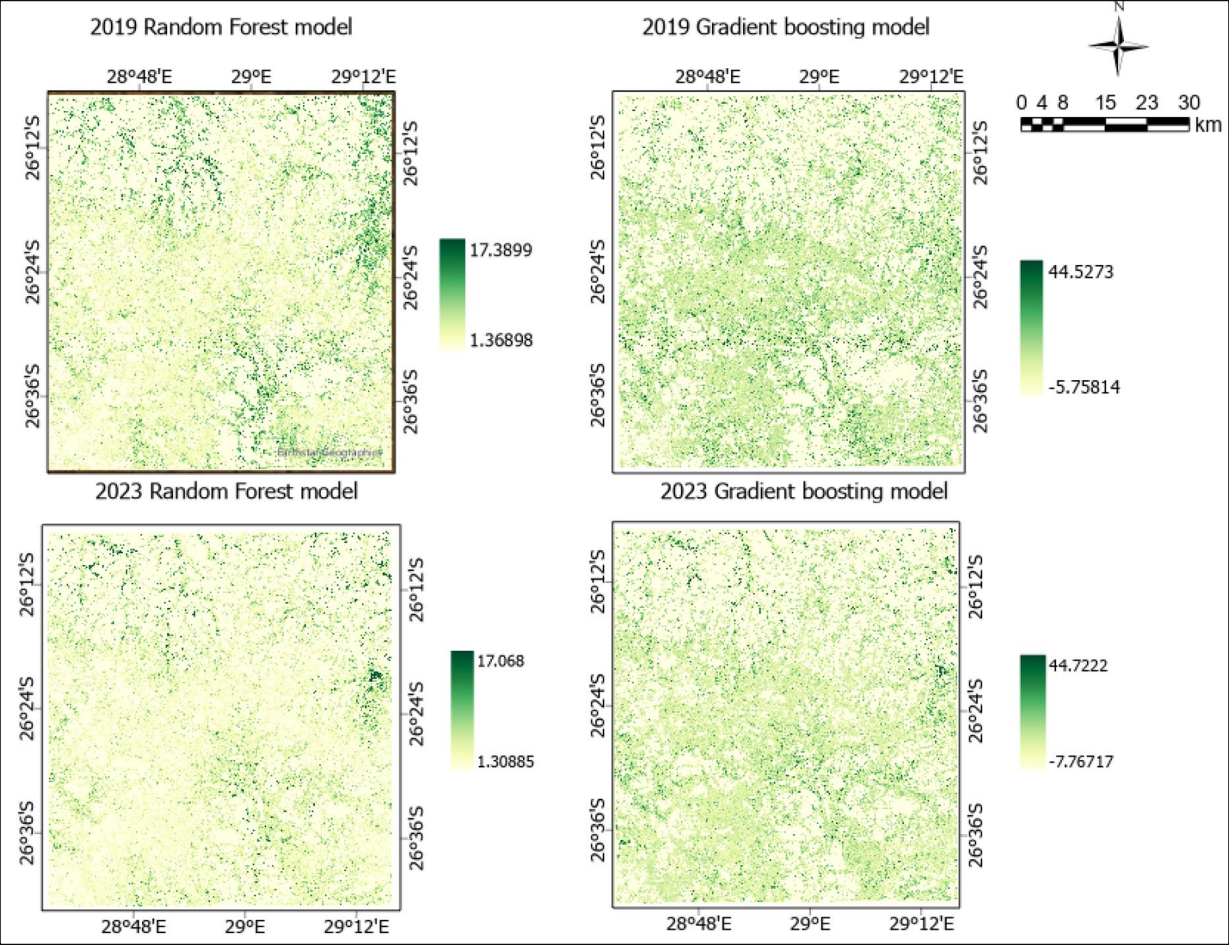
Gradient boosting and Random Forest aboveground biomass (AGB) map for 2019 and 2023

Datasets obtained from multiple sources are intergrated with the primary goal of estimating AGB. Sentinel-2 satellite imagery and SRTM derived products were used as the predictor variables, while GEDI AGBD is used as training and validation data (Fig. 4). A total of 966 AGBD data points are used with a ratio of 70% (671) and 30% (295) implemented for training and validating the model. AGB maps were produced through an integration of datasets from various sources, including Sentinel-2, GEDI, and SRTM. Sentinel−2 bands—the grassland class mask obtained from a classified image, elevation, and slope—were incorporated into the RF model as predictor variables (Fig. 4). The GEDI AGBD data is used in this research to train the model using a 70% and 30% ratio for training and validating the model. The AGB timeseries analysis comenced in 2019 and ceased in 2023. Sentinel-2 scenes used were obtained either from January or February, these time spans falls within the wet season which is also regarded as the growing season. According to the literature rainfall is expected to reach its maximum between December and February and as such vegetation is expected to thrive during this period. A major contributing factor to the selection of data collection dates is the computation of the VIs. NDVI, OSAVI, MSAVI2, and NDRE are the four distinct VIs computed and correlated to the field obtained biophysical parameter LAI. Field based LAI used accounted for 77% (33) of the overall obseved samples. The remaining 23% (10)of the data was discarded as they were labeled as outliers. for the The research assesses AGB for the years 2019 and 2023. We used scenes acquired in January and February. The rationale behind the selection of data collection dates is that this study is also focused on calculating VIs such as NDVI, and the literature indicates that the growing season for vegetation is in summer, where the rainfall is expected to reach its maximum between December and February.With the aid of the rapidly expanding technology and cloud computing industries, data processing has become seamless. A series of interconnected scripts was used within the GEE platform to download, process, and construct the model. Figure 5 shows the Gradient boosting and Random Forest aboveground biomass (AGB) map for 2019 and 2023. The unit measurement of biomass data used for this study is Mg/ha. The map generally delineates the mapped AGB of the grassland biome within the study area. Different colors show high and low AGB areas within the study area. The color scale ranges between shades of yellow and green, where very bright yellow shows a low AGB and dark green shows a high AGB. The results show the variation in the content of AGB for the prescribed period.

### AGB model performance

**Fig. 6:**
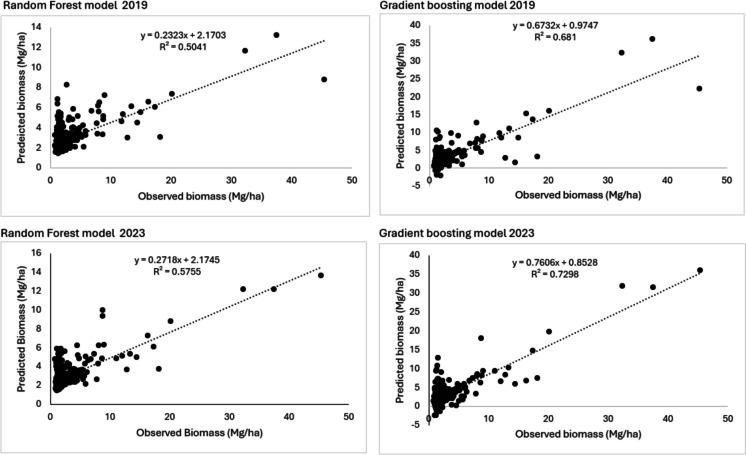
Random forest and gradient boosting regression models for 2019 and 2023

The reliability of a model is gauged by its performance and repeatability. The results depicted in Fig. 6 are used to assess the performance of the maps shown in Fig. 5. It shows the predicted biomass on the y-axis of the scatter plot and the observed biomass on the x-axis. The performance of the AGB models was assessed using the *R*^2^ parameters, which is the coefficients of determination. We used the Gradient boosting and Random Forest models to estimate AGB. The random forest model obtained an *R*^2^ value of 0.5041 Mg/ha and an RMSE of 3.42 Mg/ha in 2019 and *R*^2^ of 0.5755 Mg/ha and RMSE of 3.24 in 2023. The gradient boosting model obtained *R*^2^ of 0.681 Mg/ha and RMSE of 2.41 Mg/ha in 2019 and *R*^2^ of 0.7298 Mg/ha and RMSE value of 2.21 Mg/ha in 2023. The results show that the gradient boosting method outperformed the random forest method bases on the *R*^2^ values.

### RF variable of significance

**Fig. 7:**
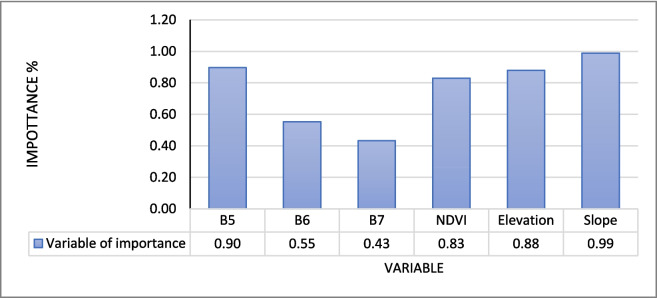
Gradient boosting variable of importance

**Fig. 8:**
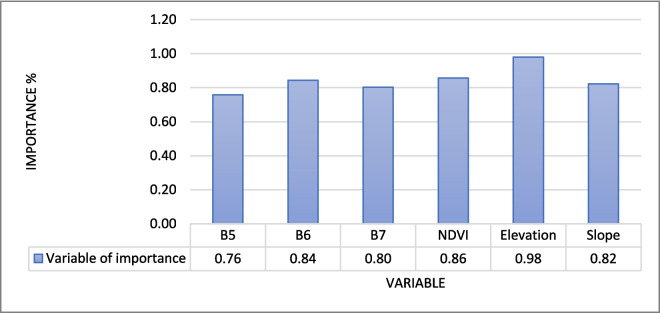
Random forest variable of importance

To determine the variables most significant in the model prediction, we used the trial-and-error method. We started off with spectral bands from Sentinel-2 satellite (B2, B3, B4, B5, B6, B7, B8, B11, and B12), elevation, and slope from SRTM DEM. The model showed signs of overfitting since some of the incorporated predictor variables had less influence in the model performance. It was then decided to remove (B2, B3, B4, B8, B9, B11, and B12) from the model, and the was a slight increase in the performance. After deciding to add a vegetation index NDVI, the model accuracy finally improved. Sentinel-2 satellite (B5, B6, B7), elevation, and slope from SRTM DEM and NDVI computed from Sentinel-2 became the final model predictors. The variables were found to be significant or relevant in predicting the models, although the level of significance varied. In the RF model of 2023, elevation was the most significant variable, and the least was Sentinel-2 band 5 (Fig. 8). Figure 7 shows the gradient boosting model of 2019, slope was the most significant variable, while band 7 of Sentinel-2 was the least.

### Independent model validation

**Fig. 9:**
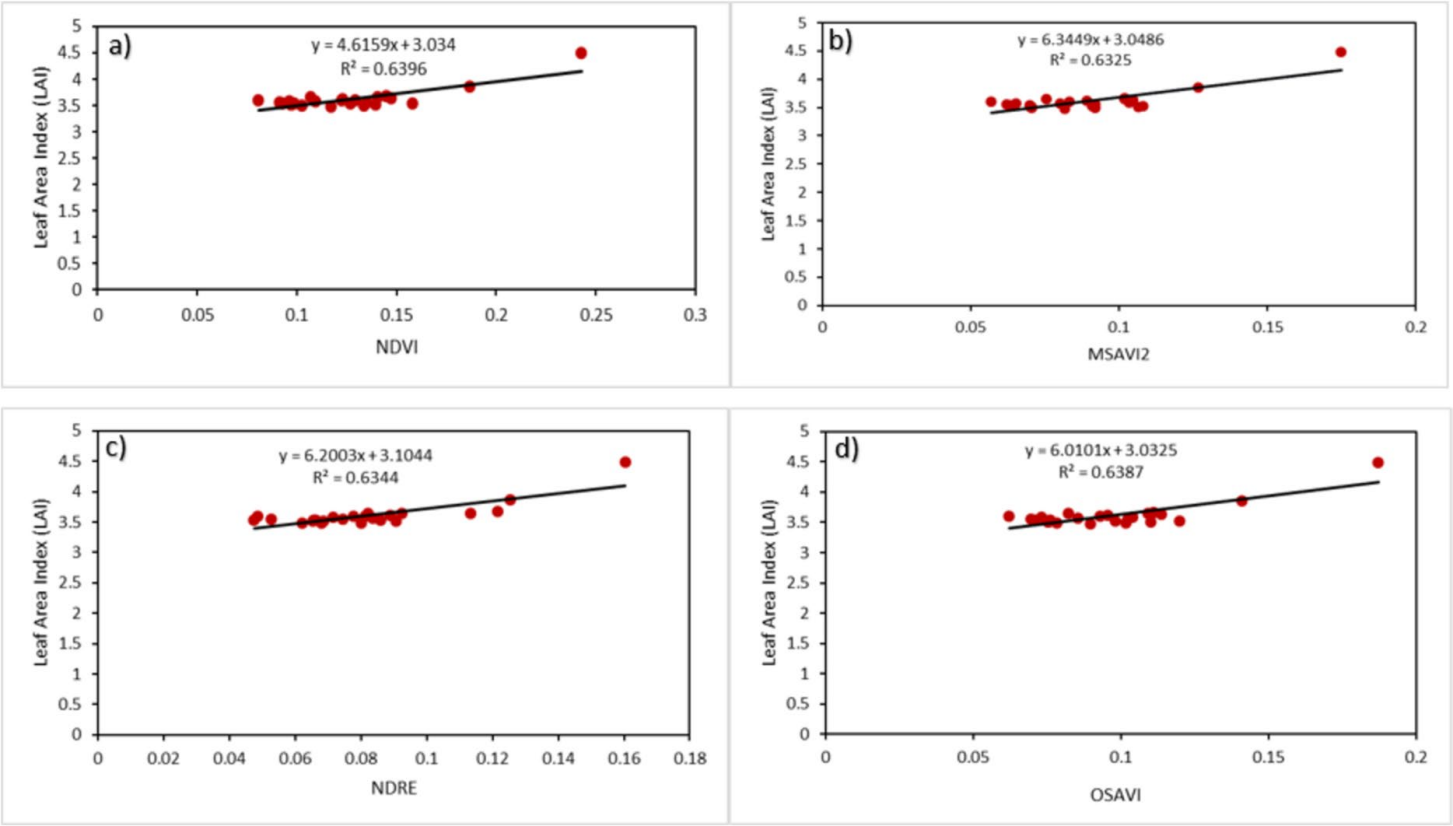
Regression model demonstrating the correlation between vegetation indices and LAI

Regardless of the perceived power, no single approach is impervious to constraints. Therefore, having a complementary approach in place is imperative. To make up for the constrains contained in random forest, we corelated independent data from Sentinel-2 (VIs) with field-based LAI data samples. One method of validation needs to be revised to declare the model credible. This research further validates the models using independent data from Sentinel-2 and the field. The independent model accuracy assessment method used VIs and a biophysical parameter. The VIs used included NDVI, MSAVI2, OSAVI, and NDRE, the LAI’s biophysical parameter. The variables in question can be utilized to quantify biomass. The objective is to assess the relationship between vegetation indices and the biophysical parameter LAI, as the latter is also utilized to quantify biomass. In the absence of the degree of accuracy, it is impossible to draw concrete conclusion concerning the model. For this reason, we adopted coefficients of determination, the *R*^2^ and the RMSE. The independent model accuracy assessment also used the coefficients of determination, the *R*^2^ and the RMSE, to determine the level of accuracy in the models. The statistics shown in Fig. 9 are indicative of well performing models, an *R*^2^ value above 60% and an RMSE value closer to zero. Although the difference is insignificant, the NDVI vs LAI model outperformed the other models explaining 63.96% of the variation in the data. OSAVI is the second best-performing model, explaining 63.87% of the variation in the data, followed by NDRE, which explained 63.44% of the variation in the data, and the poorest-performing VI is MSAVI2, which explained 63.25% of the variation in the data. According to these findings, the models are statistically significant, and a strong correlation exists between VIs and LAI.

## Discussion

Mapping the above ground biomass (AGB) of the grassland is essential, the terrestrial ecosystem provides is a home to some of the rare and endangered species and plays a fundamental role in the water cycle (Netsianda et al., 2024). The findings of this study provide evidence that it is indeed possible to map AGB of the grasslands, thereby accounting for carbon stock residing in this terrestrial ecosystem. Two ensemble approaches the gradient boosting and RF models successfully mapped the AGB of the grassland. RF and gradient boosting both use regression trees to train the model; however, they are different in that the former employs a splitting function and uses a random selection, while the latter uses all the regression trees to train the model. In their study, Banerjee (2022) showed that the two methods can achieved *R*^2^ values that are statistically significant. While Banerjee (2022)’s findings are not different from those of the current study, RF and gradient boosting were able to predict the biomass achieving *R*^2^ that is above 0.5. This means that the models were able to explain more than 50% of the variation in the data in all models. Yu et al. (2021) stated that an *R*^2^ value that is high and RMSE value that is closer to zero indicates a good model. Both algorithms were trained using the same amount of data (GEDI AGBD) and predictor variables (Figs. 7, 8 and 9). The GEDI AGBD data possesses challenges related to geolocation faults. However, this issue was reduced by filtering the data. The RF and gradient bosting algorithms showed ability and capacity to handle multi-modal data and discern between good and bad data. Sentinel-2 bands (B2, B3, B4, B8, B11, B12) were eliminated from the model as they introduced noise (Nasiri et al., 2022). In a study conducted by Banerjee (2022), RF outperformed gradient boosting; however, the current study reveals the opposite. Although gradient boosting outperformed RF, the findings of the latter are no different from those presented in research conducted by Naidoo et al. (2019) for estimating AGB in the vegetated wetlands of the grassland biome in South Africa using the RF technique. Their findings demonstrate that the models were statistically significant, achieving an *R*^2^ value of 0.63 and an RMSE of 169.28 g/m^2^. In the current study, RF achieved the highest *R*^2^ value of 0.5755, while gradient boosting achieved the highest *R*^2^ value of 0.7298. The processing duration was significantly reduced, all thanks to the GEE platform for its robust computational powers. GEE also provided easy access to multiple datasets, which enabled a successful fusion thereby successfully estimating the AGB of the grassland biome.

Although the RF AGB model could assess its accuracy using the *R*^2^ and the RMSE parameters, it is equally important to consider other ways of validating the model to ensure reliability and repeatability. This research took the initiative to use independent datasets obtained from Sentinel-2 and the field to validate the RF AGBD model further. It used the VIs and the biophysical parameter (LAI) information. The VIs used are NDVI, MSAVI2, OSAVI, NDRE, and the biophysical parameter LAI. Previous studies have indicated that information obtained in the red, red-edge, green, and near-infrared bands correlates well with LAI (Masemola et al., 2016). Pang et al. (2020) also indicated the significant correlation that exists between these indices and biomass. Since LAI and NDVI are related to biomass, they can be used as independent data to assess the accuracy of the RF and gradient boosting AGB model. This information is supported by results obtained by Masemola et al. 2016 who used Landsat 8 sensor band information and field-based measurements of LAI to establish the correlation between various VIs and LAI. Their research findings showed that there is a correlation between VI and LAI. NDVI vs LAI linear correlation achieved an *R*^2^ value of 0.38 and 0.36 and an RMSE value of 0.31 and 0.50. The current study also followed a similar approach, and the findings indicated that the VIs vs LAI models were statistically significant, with an *R*^2^ value above 0.6 for all the models between VI and LAI. Although NDVI is known for being sensitive to terrain not entirely covered by vegetation, it outperformed the other VIs. According to Wang et al. (2022), this outcome is contradictory because we expected VIs with the soil adjusted factor to perform better since they can overcome the limitation of NDVI. The findings revealed that the VI vs. LAI models were statistically significant, with an *R*^2^ value greater than 0.6 for all models. The results show that Sentinel-2 bands, vegetation indices, and SRTM DEM products are valuable tools for quantifying biomass. Despite limited financing, scarcity of validation datasets, restricted access to certain survey areas, and unpleasant weather conditions, this research successfully achieved its goals.

## Conclusions

This study was based on the use of RF and gradient boosting machine learning algorithms known for their ability to handle a multi-modal of datasets and high computation power. The objective of mapping the AGB of the study area was achieved. Fusing multiple datasets such as slope and elevation, Sentinel-2 sensor bands, vegetation index (NDVI), grassland mask derived from a classified map, and training datasets GEDI mean AGBD was practical in successfully estimating AGB, thereby providing valuable information about carbon stock. We also established that ensemble algorithms are valuable tools in predicting AGB. Their findings were statistically significant. The use of field LAI data and vegetation index data extracted from the Sentinel-2 sensor to validate the model was beneficial because the validation models proved to be significant (with *R*^2^ values above 0.6). The NDVI model outperformed the rest, obtaining the highest *R*^2^. Using various datasets and methodological approaches, this research has successfully managed to monitor vegetation dynamics in the study area. The results show variations or changes in vegetation biomass over time. The current study focused on the grassland biome without considering different species or categories of grasses. Although the data used was sufficient to validate the AGB model, future studies are recommended to establish the location of different species of grasses and distribute the field samples among selected grass types/species instead of simply randomly sampling. This might improve the model by ensuring that ground sampling points fairly represent grassland species. It is recommended to include other machine learning algorithms to compare with ensemble methods. Considering the remarkable performance of the ensemble machine learning algorithms in the current study area, it is worth considering the application of this approach in other grassland areas with varied climate conditions and on a broad scale. This model can also be implemented by changing a few parameters to estimate forest carbon.

## Data Availability

No datasets were generated or analysed during the current study.

## Declarations

All authors have read, understood, and have complied as applicable with the statement on “Ethical responsibilities of Authors.”
